# Nursing practices in the primary health care context: a scoping review**^1^**

**DOI:** 10.1590/1518-8345.0880.2721

**Published:** 2016-08-29

**Authors:** Rosangela Barbiani, Carlise Rigon Dalla Nora, Rafaela Schaefer

**Affiliations:** 2 PhD, Adjunct Professor, Universidade do Vale do Rio dos Sinos, Porto Alegre, RS, Brazil.; 3 Doctoral Student, Instituto de Ciências da Saúde, Universidade Católica Portuguesa, Porto, Portugal.

**Keywords:** Nursing, Primary Health Care, Nurse's Role, Nursing Care

## Abstract

**Objective:**

to identify and categorize the practices performed by nurses working in Primary Health Care and Family Health Strategy Units in light of responsibilities established by the profession's legal and programmatic frameworks and by the Brazilian Unified Health System.

**Method:**

a scoping review was conducted in the following databases: LILACS, IBECS, BDENF, CINAHL and MEDLINE, and the Cochrane and SciELO libraries. Original research papers written by nurses addressing nursing practices in the primary health care context were included.

**Results:**

the review comprised 30 studies published between 2005 and 2014. Three categories emerged from the analysis: practices in the service; practices in the community; and management and education practices.

**Conclusion:**

the challenges faced by nurses are complex, as care should be centered on the population's health needs, which requires actions at other levels of clinical and health responsibility. Brazilian nursing has achieved important advancements since the implementation of policies intended to reorganize work. There is, however, a need to shift work processes from being focused on individual procedures to being focused on patients so that an enlarged clinic is the ethical-political imperative guiding the organization of services and professional intervention.

## Introduction

The Brazilian Unified Health System (SUS) is a public policy designed in the VIII National Health Conference that was constructed and institutionalized after a broad debate in Brazilian society that was encouraged by the health movement and was partially upheld by the Federal Constitution of 1988. It is a social experiment, the advancements of which are unquestionable, though considerable challenges still remain^1^. Brazil is the only country with more than 100 million inhabitants with a public health system that has universal coverage and that provides integral care free of charge - these are characteristics, which coupled with the country's continental dimensions, demographic transitions and epidemiological features, in addition to regional inequalities, entail many challenges to the system's consolidation.

One of these challenges arises from the health situation in Brazil, which has changed and is currently characterized by accelerated demographic transition, but which expresses a triple load of diseases - there is an unmet agenda of infectious and deficiency diseases, diseases caused by external causes, and the predominant presence of chronic conditions. Altogether, this synthesizes a situation that cannot be given a proper response by a still very fragmented, reactive, and episodic health system, mainly focused on coping with acute conditions and acute exacerbations of chronic conditions, in which the hospital is the privileged locus of the care model^1^.

Primary health care (PHC), a strategy to cope with this context and to support SUS, has gained recognition and increasing responsibilities as it is the entrance door into the system and the junction that connects and coordinates the healthcare networks. The PNAB (Primary Care National Policy), established in 2006, was recently updated^2^ to expand the coverage of services, programs, and territories to meet emerging health needs and demands. The policy is guided by principles of universality, accessibility, establishment of bonds, continuity of care, integrality of care, accountability, humanization, equity, and social participation; that is, the guidelines of the new health care model implemented by the SUS are the guiding principles.

Measures to promote health, prevent disease and improve access to the system should have priority at the PHC level, especially via the Family Health Strategy (FHS), through which it is possible to reach areas and regions with greater population coverage. In the scope of health care delivery, the increase of chronic and complex diseases and the rapid aging of the population, have also led to significant increase in the number of visits of patients to PHC services. It is worth noting that there were approximately 30,000 family health teams providing care to about 98 million people in 2010^3^. In this sense, systems around the globe have invested in remodeling healthcare actions to cope with the high costs of intermediate and highly complex services and the low capacity of interventions to solve health problems^4^ ^-^ ^5^.

The role of nurses, be they in a management position and/or delivering care, providing education or promoting preventive measures at the PHC level, is essential and strategic. For this reason, the SUS, through its programmatic and legal frameworks, has ensured the presence of nurses on staffs and in covered areas^2^. Despite the positive aspects accruing from the reorganization of the care model, socio-occupational demands imposed within this space of intervention are complex and lead to dilemmas and ethical issues, theoretical-methodological and technical-operational issues inherent to the profession. The reason for this is that the demands faced in the routine of services still reflect the biomedical model, in which care is generally provided in a hospital setting through technical procedures and therapeutic diagnoses^6^. Contrary to practices guided by this model, attentive listening, reception, bonding, and shared liability under the logic of an enlarged clinical practice, as well as matriciamento* and interdisciplinary and inter-sector interventions addressing social determinants of health, all constitute examples that require innovative work processes.

As a consequence, a paradigm shift is underway and for it to be consolidated, scientific research can contribute by disseminating experiences and conducting investigations, in addition to the systematization of what has been published in Brazil regarding nursing interventional agendas and its instrumentalities. Therefore, considering the complexity of requests imposed on nurses at the PHC level, this study's objective was to identify and categorize the practices performed by nurses in PHC units and Family Health Care Units, in light of the responsibilities established by the legal and programmatic frameworks of the profession and the SUS.

The practices expected from nurses in the PHC context are clearly described in legal documents that govern the profession and the health system. In this study, however, we intended to compare what is provided in legal documents and the nurses' actual professional practices; that is, investigation, care, and public health policies were compared. This will lead to the problematization of practices and corresponding theoretical and ethical assumptions to achieve the results expected by nursing, enabling a critical and propositional debate concerning the profession's contributions and limitations. It is important to emphasize that one of the positive aspects of this study is the breadth of the analysis of studies conducted in diversified and unique contexts in Brazil, with the potential to support policy decision-making, health leaders and the nurses themselves who can use knowledge to strengthen nursing and PHC^7^.

## Method

The scoping review, a methodology described by Arksey and O'Malley^8^ and that was later systematized^9^, was adopted for this study. Even though a scoping review shares various characteristics of a systematic review, such as methodical, transparent and replicable aspects, studies adopting the first are designed to obtain less depth but broad and comprehensive results^10^.

This scoping review is intended to assess and clarify the state of knowledge concerning nursing practices performed in PHC services based on the results of empirical studies comparing reality with the underlying theory. The scoping review's six methodological steps were followed: (1) identify the research question; (2) find relevant studies (search for relevant studies); (3) select studies; (4) extract data; (5) separate, summarize and list the results; (6) report results^8^ ^-^ ^9^. The form used for this review is described in the study by Levac, Colquhoun and O'Brien^9^.

The research question should be open in order to reach the desired range of responses. A clear purpose, combined with a well-defined research question, enables researchers to achieve more accurate conclusions and eases the selection of studies and the extraction of data^8^. The question established for this study was: what is it known about the practices of nurses in the different PHC services in Brazil?

To ensure the identification of the most relevant studies addressing this topic, the search strategy should consider the terms to be used, sources to be searched, period of time, and the language of the papers^10^. Therefore, to properly answer the study's question, we opted to search primary studies describing the contexts of the practices performed by nurses published in indexed sources or grey literature written in Portuguese. 

The search included studies published from 1988, when the Federal Constitution was ratified, to December 2014. In order to be comprehensive, various sources were consulted, including the following databases: Latin American and Caribbean Health Sciences (LILACS); Spanish Bibliographic Index on Health Sciences (IBECS); BDENF (Nursing Database); Cumulative Index to Nursing and Allied Health Literature (CINAHL); and Medical Literature Analysis and Retrieval System Online (MEDLINE); the Cochrane Library; the Scientific Electronic Library Online (SciELO); and Google Scholar, in addition to lists of relevant literature references. The search terms were related to components of nurses' practices and the context of nursing work, including nursing, nurse, nurse practice, basic health care, primary health care, family health strategy, family health team, community health, and public health.

Clearly defined exclusion and inclusion criteria are essential to selecting the studies^10^. Excluded studies were those addressing subjects other than nurses, such as students, patients or other workers, studies conducted in contexts different from that of primary health care, such as hospitals or teaching facilities, or, finally, studies conducted in countries other than Brazil. To be eligible, studies should address nurses in the primary health care context and topics concerning the practice of nurses. Two researchers independently conducted the search between September and December 2014. Disagreements were discussed with a third researcher and resolved by consensus that is, results from the independent search conducted in the databases were compared and differences were verified in order to comprise the highest number of studies addressing the topic.

The mapping of data with the use of a structured instrument enabled the identification of the studies' essential elements, which allowed the synthesizing and interpretation of data and the generation of a basic numerical analysis of the extension, nature and distribution of studies included in the review. Finally, the results were compiled to present an overview of the overall content through an organized thematic construction in accordance with the nature of nurses' practices in the PHC context.

## Results

A total of 30 papers, published between 2005 and 2014, were found; most were published in 2011^11^ ^-^ ^19^. The papers were published in Brazilian journals classified between A1 and B3, according to the Qualis system (CAPES). Most studies were conducted in the South (n=10) and Southeast (n=13) or 76.6% of the sample; followed by the Northeast with four studies (13.3%); Midwest with two papers (6.6%); and finally, one paper was conducted in the North of Brazil (3.3%).

All the studies addressed nurses working in PHC services, as this was an inclusion criterion. Nineteen studies addressed nurses who exclusively worked in FHS; four addressed nurses working in PHC units; two papers addressed nurses from the PHC Network; and one study addressed nurses from each of the following services: Extra-hospital services, Family Health Unit, and Municipal Health Center. There were also two studies that concomitantly interviewed nurses from different services, such as FHS and PHC Units, and nurses from the Municipal Health Center and a PHC unit. A total of 479 nurses composed the data set.

Additionally, only one study, out of the 30 studies analyzed, adopted a quantitative methodology and also interviewed nurse managers^20^. Its data were collected through interviews and analyzed using EpiData. The remaining studies used qualitative methods: either interviews (n=24; 80%), questionnaires (n=2; 6.6%), observation (n=1; 3.3%), or focus groups (n=1; 3.3%). Another two studies combined interviews and observation (n=1; 3.3%) and interview and document analysis (n=1; 3.3%). Among the qualitative analysis methods, the most frequently used was content analysis (n=14; 46.6%), followed by thematic analysis (n=6; 20%), and discourse analysis (n=5; 16.6%). The hermeneutic, cartographic, and categorical analyses and analysis based on grounded theory appear only once each (3.3%).

The reading and analysis of studies enabled the identification of the nurses' practices. The findings were thematically organized according to the nature of their practices, which were classified into practices in the service, practices in the community, and management and education practices. 

### Practice in the service

The category practice in the service comprises the actions of nurses preferably - or mainly - performed within the health services (Figure 2), though it does not exclude such actions being performed within the community, such as nursing consultations, procedures and health promotion actions.

**Figure 1:**
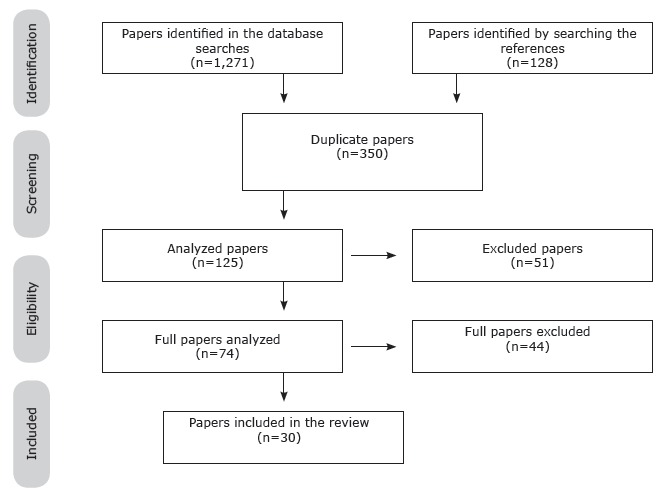
Selection of studies in the databases

**Figure 2:**
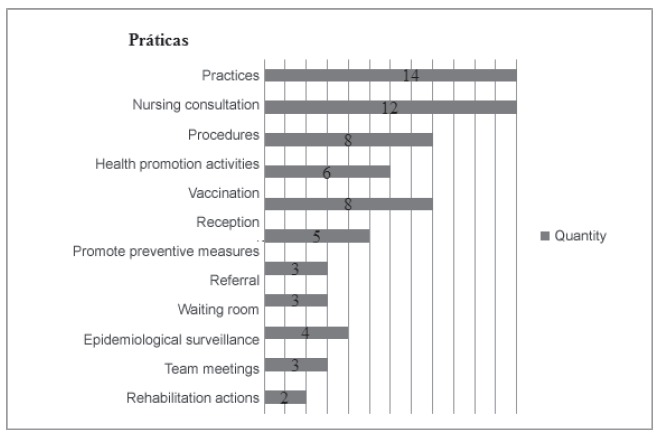
Practice in the service

The practice of nursing consultations stood out among the studies^11^ ^,^ ^14^ ^-^ ^15^ ^,^ ^19^ ^-^ ^29^. Consultations are used by nurses to identify patients' needs, which (re)establishes priorities of health actions^24^. Other activities developed by nurses were technical and educational practices. The technical practices mentioned in the studies included applying dressings^14^ ^,^ ^20^ ^,^ ^23^ ^-^ ^24^ ^,^ ^28^ ^,^ ^30^ ^-^ ^31^, measuring blood pressure^14^ ^-^ ^15^ ^,^ ^20^ ^,^ ^23^ ^-^ ^24^ ^,^ ^28^ ^,^ ^30^ ^-^ ^31^, verifying blood glucose^13^ ^,^ ^23^ ^,^ ^31^, the heel prick test^13^ ^,^ ^23^, cytology collection^15^ ^-^ ^16^, requesting tests^23^ ^,^ ^32^, examination to prevent breast cancer, insertion of urinary catheters and nebulizers^23^, preventive measures^33^, verification of anthropometric and nutritional measures^26^, application of injections^24^ ^,^ ^28^, delivery of medications^31^, administration of medications^21^, and assessment of laboratory exams requested by physicians^23^.

Educational actions are directed to specific population groups, such as children, adolescents, adults, women, mental patients, diabetic, hypertensive, and individuals with tuberculosis, among others^11^ ^,^ ^15^ ^-^ ^16^ ^,^ ^20^ ^,^ ^23^ ^-^ ^24^ ^,^ ^34^ ^-^ ^35^. Other practices performed by nurses working in the PHC context include clinical care^36^, attending to urgent and emergencies situations^35^, supporting medical care^11^, providing prenatal care^15^, and assessing risk classification^27^.

### Practices in the community

This category includes actions nurses perform outside the health unit, though these activities can also be performed within the health services. 

**Figure 3:**
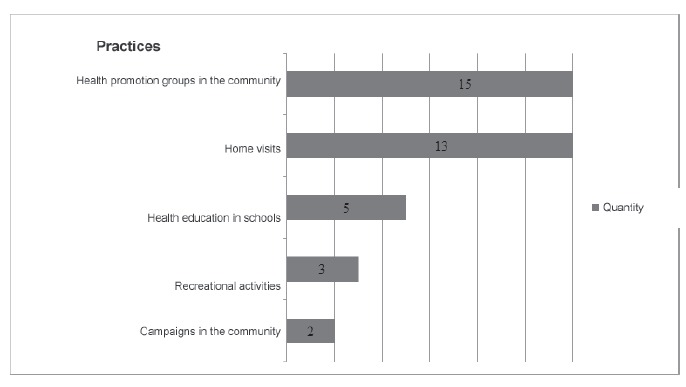
Practices in the community

The health promotion groups aggregate the practices that were most frequently found in the studies^11^ ^,^ ^13^ ^,^ ^15^ ^-^ ^16^ ^,^ ^18^ ^,^ ^23^ ^-^ ^28^ ^,^ ^30^ ^,^ ^34^ ^,^ ^37^ (Figure 3). One study describes group activities as supporting patients in periods of change, of treatment or crises, by helping them adapt to healthier behaviors^31^. Some studies, however, report the traditional approach to transmit knowledge in the form of a vertical dialogue^23^ ^,^ ^30^ ^-^ ^31^. The group activities are directed to pathologies or specific conditions - such as hypertension, diabetes, asthma, mental health and tobacco^23^ ^,^ ^31^ - or specific populations, such as pregnant women, children, the elderly, and those receiving Bolsa Família (social welfare program); these are some of the activities performed in the services^15^ ^,^ ^23^ ^,^ ^31^.

Home visits were also one of the practices most frequently reported by studies^11^ ^,^ ^12^ ^,^ ^14^ ^,^ ^17^ ^,^ ^19^ ^,^ ^23^ ^,^ ^27^ ^-^ ^30^ ^,^ ^38^ ^-^ ^39^. This strategy is used to bring the health team into proximity with the families and community^13^ and to enable nurses to identify the context(s) in which they have to perform and then to be included in a given community. The delivery of integral nursing care is possible when a horizontal relationship is created by the establishment of bonds^12^. Therefore, home visits are a key instrument for nurses working in PHC.

The main purpose of health education activities promoted in schools is to encourage preventive measures addressing oral hygiene, drugs, sexuality and unplanned pregnancy^15^ ^,^ ^18^ by using recreational activities^40^.

### Management and education practices

**Figure 4:**
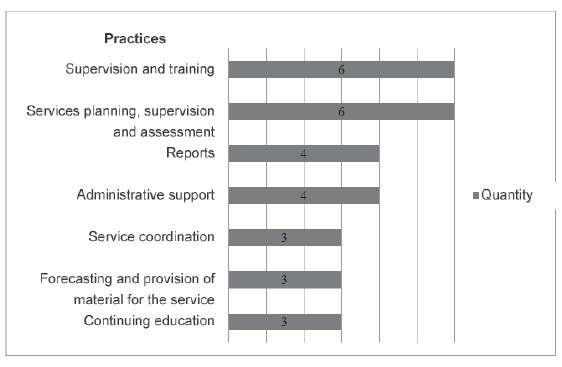
Management and qualification practices

The category management and education practices presents and characterizes the coordination and management actions carried out by nurses in the scope of PHC (Figure 4). Among management practices, planning is described as essential to developing an action plan for activities that will be performed by nurses in the routine of health services^36^, with an emphasis on the idea that nurses should be able to simultaneously perform care and management activities^35^. The range of routines that unfolds from these attributions is associated with the notion of ethical competencies. Nurses construct technical and scientific knowledge during academic education to ensure a broad view concerning the health/disease continuum, taking advantage of this knowledge in their daily practice, with autonomy and the competence to perform tasks and the understanding to maintain respectful behavior toward their team members^13^.

Nurses from the basic health care network are responsible for supervising and training nursing technicians and/or auxiliaries^11^ ^,^ ^23^ ^-^ ^24^ ^,^ ^36^ ^)^ and health community agents^11^ ^,^ ^19^ ^-^ ^20^ ^,^ ^29^ ^,^ ^36^ ^,^ ^40^, in addition to conducting continuing education activities with workers^14^ ^,^ ^40^. The studies also include practices that involve the participation of nurses in Local and City Health Councils^36^, technical responsibility for the Regional Nursing Council^40^, fundraising, integrating and promoting a good relationship with the health staff, information system recording^35^, hiring and training human resources^15^, organizing schedules of days off and vacation^23^, scheduling consultations with specialists^23^, reporting diseases^23^, and recording the team's production^13^.

## Discussion

The results show considerable variation in the number of studies per region; only one, out of the 30 studies included in the review, was conducted in the North. The different geographic regions of the Brazilian territories present very distinct demographic, economic, social, cultural and health conditions, with considerable inequality^3^. The North is considered one of the poorest regions in Brazil, while the Southeast is responsible for 56% of the gross domestic product^3^, which may indicate the northern area requires greater attention be paid to health issues so that the low number of studies conducted in the North would represent this inequality. Another explanation for the difference in the number of publications per region may be related to the number of existing graduate programs. According to the study by Erdmann, Fernandes and Teixeira^41^, the North has only one graduate nursing program, while the Southeast totals more than 19 graduate programs.

The creation of the SUS represented an important advancement toward the improvement of the supply of and, more importantly, access to health services in Brazil. The creation of the Family Health Program and its further transformation into strategy, as well as updating the PNAB^2^, consolidated this advancement and broadened the conception of health, aiming to promote the delivery of integral care^3^. In this sense, the greater incidence of studies focusing on PHC services implemented within the FHS shows that its objective to function as a reference for decentralized care and the preferred contact of patients, has been achieved.

In regard to the nurses' interventional agendas reported in the studies concerning the attributions provided by the PNAB^2^, these responsibilities are partially met because those specific to care delivery predominate, among which nursing consultations, procedures and group activities, requests for complementary exams, the prescription of medications, referral of patients to other services, implementation of programed activities and delivering care to spontaneous demands.

In this context, the fact that the composition of FHS teams is limited at the same time demand is increasing may lead professionals to prioritize necessary but routine and less complex tasks, tasks that demand much less complexity than a nurse's potential competence. From this perspective, the enlarged clinic is presented as a tool for health work processes to deliver patient- centered care in order not only to address the disease, but also the individual within his/her context and at the collective level^2^.

The practices of nurses concerning health care delivered in the remaining spaces within the community-namely, planning, managing and assessing the actions performed by the health community agents, contributing, participating and implementing continuing education activities and managing the inputs necessary to the health unit's proper functioning-were less frequently mentioned in the interventional agendas compared to the traditional ones.

The practices shared with the remaining team members, such as ensuring that integral health care is delivered, and promoting programed and collective actions in addition to health surveillance, among other activities, also appeared occasionally in the nurses' work processes. Another study^42^ discussed this context, showing that nurses' work is linked to technical actions focused on direct curative care with an overload of work, while what is expected within the PHC context is a work process guided by integral care. On the other hand, home visits - one of the main activities that give nurses the opportunity to establish and strengthen bonds with the community from the perspective of integral care - are among the most frequently mentioned, concerning the practices performed within the community. Note this is a complex activity and when it is well performed, it can impact the determinant factors of the health/disease continuum and enable the family to acquire greater autonomy in the process of health production^43^.

The results of this review reveal the same difficulties faced in the international context, specifically in Portugal, where difficulties concerning the professionals' work processes, such as issues related to the non-compliance of schedules, failures in the information system, lack of administrative autonomy to buy supplies, and political-institutional uncertainty, are also experienced(44). Additionally, the same study reports that home visits present some weaknesses, such as a predominantly clinical-curative approach and care for patients with impaired mobility.

The educational actions promoted by nurses were listed both in the category practices in the service and the category practices in the community. Health education in both contexts is promoted through programed actions designed for specific groups based on disease, age or gender, a strategy that does not favor the recognition of each patient's unique needs and those of the community in general, while the delivery of integral care practices is not promoted, either^42^.

The role of nurses in educational processes should give priority to participatory actions, avoiding restricting care to a curative logic, but rather promoting integral care models and humanization^42^. Another indicator of this trend is underlined in the non-expressive participation of nurses in actions promoted in schools, despite the SUS' guiding policies. The Programa Saúde na Escola [School Health Program], established jointly by the Ministry of Education and Ministry of Health, is an example: it is implemented in 87% of Brazilian municipalities and is put into operation by adjusting actions that integrate PHC teams and/or family health strategy teams and public schools^45^.

The management practices category presents results that put nurses in a role in which they administer care. Nurses develop care and management actions, often simultaneously, so that these professionals are seen as multipurpose individuals in the team. According to a study^46^, management actions should be jointly performed by the entire health team; however, the results show that nurses report various activities related to the management of routines, suggesting there is a weakness at this level of care and a rupture with care delivery. In this approach, another study^43^ verifies that nurses are unsatisfied with work overload, as they perform activities that are a responsibility of other workers, which harms the delivery of integral care. Therefore, this shows a challenging reality in which fragmented care centered on the individual/patient should be overcome. Despite notable advancements, traditional practices concerning the health/disease continuum, and their underlying rationale, still persist among nurses working in the PHC network. In other words, there is a reproduction of interpretative and interventional instruments focused on the health/disease continuum^5^ ^,^ ^46^.

This context is not exclusive to nurses. Studies indicate that, whether due to a lack of knowledge of the system or to a view directed to individual health needs, managers, workers and patients impose restrictions on experiences of an educational or preventive nature, intensifying contradictions between SUS principles and the way the network is managed and work processes^5^ ^,^ ^47^. Hence, even though many are considered humanizing practices, changes are not achieved in the health services due to a lack of a deeper analysis of work processes and continuing education. 

Given the contradictions existing between the Brazilian population's health needs and the persistence of attitudes and practices that resist changes being implemented in the model, the SUS has implemented policies and programs that affect matters from formal education up to the organization of services. In this context, the responsibility of nurses working in the PHC network increased considerably^47^, especially those attributions directed to health promotion and continuing education.

In addition to the care delivery and management responsibilities common to any nurse working in the PHC network, nurses in the FHS should contribute to the organization of health care, qualification of access, reception, establishing bonds, care over time, and guide the work performed by the staff in PHC units given the priorities equitably established in accordance to health needs, vulnerability, and risks, among other aspects(2). In order to consolidate this model, the SUS has designed projects and established regulations concerning the organization of services, formal education, in-service education, or by assigning new roles and responsibilities to healthcare workers, as is the case of the DCN (National Curriculum Guidelines)^48^, established in 2001, which prescribe guidelines for the preparation of curricula of programs in all Brazilian Higher Education Institutions (HEI) that offer instruction in professions in the health field (nursing, medicine, and nutrition).

Article 5 of the DCN establishes that the education of nurses should meet social health needs, emphasizing the SUS and ensuring integral, quality and humanized care^48^. In this context, nursing is challenged to seek paths that critically and effectively respond to the health issues presented by society. The ethical-political and technical-operative posture underlying this call for practices aligned with the SUS is confirmed by the profession's Ethical Code^49^, as it prescribes that nurses should take part in activities intended to meet the population's health needs and to defend the principles contained in public and environmental health policies, encouraging universal access to health services, integral care, problem-solving capacity, patient autonomy, the participation of the community in health-related issues, and the health services' political-administrative hierarchization and decentralization.

The identified gaps refer to two interconnected levels that impact the professional practice of nurses. The first, the relative dominance of new management technologies, refers to underlying concepts that guide the organization of the work process in PHC care ^(^ ^2^. This operational gap in the scope of nurses' work processes reveals the second critical node revealed in the results, which refers to the formal education of nurses and the need to problematize professional knowledge/practice in light of these new conceptual and methodological references.

Discussions concerning DCN guidelines established for undergraduate nursing programs have mobilized professionals, confirming the strength and relevance of this debate that needs to gain attention in the graduate context, at the academic and professional levels, to produce changes in the service practices over the short term, emphasizing work processes.

In this direction, various initiatives have been proposed, such as the implementation of a Nursing Residency Program focused on PHC and the inclusion of advanced nursing practices, especially in professional Master's programs ^50^. These initiatives are intended to enlarge the scope of the practices of nurses, developing and deepening the work among different professions in the PHC to achieve greater problem-solving capacity^51^.

## Conclusion

This review's results show that, even though PHC is expanding, the challenges faced in the implementation of the principles that guide PHC are complex, because they beckon to another care model, centered on the population's health needs, which leads to actions implemented at other levels of clinical and health responsibilities.

This review presents some limitations. The initial purpose was to assess most of the existing literature. The possibility of accomplishing this, however, is limited, as there may be studies published in other languages and in index databases not included in this study.

The conclusion is that this review's results are useful for future studies addressing the practice and education of nurses. The synthesis of the results reported by studies conducted in Brazil eases the incorporation of scientific relevance into practice; that is, it allows transferring knowledge to nurses as they can identify their practices performed in PHC and FHS units. Thus, a difference in the delivery of care can be achieved by linking evidence-based and practice-based knowledge.

The results show that the nurses' interventional agendas are being transformed in the dialectic of ruptures and continuities, sometimes updating old polarizations (care and management, occasional and programmatic care), and sometimes requiring and promoting innovation, beginning with the clinic of care up to actions that qualify access, promote health, and health and continuing education, in accordance with PNAB's guidelines.

The challenges presented require that nurses contribute to the consolidation of the SUS care model, that is, instead of a work process centered on procedures and professionals, a work process centered on patients in a way in which broadened care is guided by an ethical-political imperative concerning the organization of service provided and professional intervention. Finally, changing this context, in terms of education and work processes, represents an ongoing challenge for all healthcare workers, especially nurses, given their expressive and strategic role in the health system, including PHC.
